# Knowledge and attitudes of doctors towards e-health use in healthcare delivery in government and private hospitals in Northern Uganda: a cross-sectional study

**DOI:** 10.1186/s12911-015-0209-8

**Published:** 2015-11-04

**Authors:** Geoffrey Tabo Olok, Walter Onen Yagos, Emilio Ovuga

**Affiliations:** Department of Computer Science, Faculty of Science Gulu University, P.O. Box 166, Gulu, Uganda; Department of Library and Information Service, Faculty of Medicine Gulu University, P.O. Box 166, Gulu, Uganda; Department of Mental Health, Faculty of Medicine Gulu University, P.O.Box 166, Gulu, Uganda

**Keywords:** E-health, Attitude towards e-health, ICT skills, E-health attributes, E-health use, Hospitals in northern Uganda

## Abstract

**Background:**

E-health is an essential information sharing tool in healthcare management and delivery worldwide. However, utilization of e-health may only be possible if healthcare professionals have positive attitudes towards e-health. This study aimed to determine the relationships between healthcare professionals’ attitudes towards e-health, level of ICT skills and e-Health use in healthcare delivery in government and private hospitals in northern Uganda.

**Methods:**

Cross-sectional survey design was used. Sixty-eight medical doctors in three government hospitals and four private hospitals in Northern Uganda participated in the study. A pretested self-administered questionnaire was used to collect the required data. Data was analysed using SPSS software Version 19.

**Results:**

Out of the 68 respondents, 39 (57.4 %) reported access to computer and 29 (48.5 %) accessed Internet in the workplace. Majority of healthcare professionals had positive attitudes towards e-health attributes (mean 3.5). The level of skills was moderate (mean 3.66), and was the most important and significant predictor of ICT use among healthcare professionals (*r* = .522, *p* < .001); however, attitudes towards e-health attributes did not contribute significantly in predicting e-health use.

**Conclusions:**

The findings suggest need for hospitals managements to strengthen e-health services in healthcare delivery in Northern Uganda.

## Background

E-health is defined as the use of Information and Communication Technologies (ICTs) in support of health to improve the efficiency and effectiveness of healthcare management and delivery [1]. E-health improves health surveillance, health system management, health decision making, standardised sharing of health information; and promotes equity in healthcare delivery [2–5]. E-health has potentials to improve access to healthcare and could effectively reduce professional isolation and improve healthcare worker retention in resource constraint environments [6]. Opportunities for online health education, expanding the scope of healthcare delivery, health compliance, follow-up and appointments are additional benefits of e-health [7]. E-health is an essential requirement for success in healthcare management and delivery worldwide [8, 9], and it enhances healthcare professionals’ access to information to optimize health interventions outcomes [10]. However, the effective utilization of e-health tools may be possible if healthcare professionals have positive attitudes towards e-health and they possess the skills to use information and communication technology tools. Although e-health has the potential to improve the efficiency and effectiveness of healthcare management and delivery, it has been reported that acceptance of e-health among healthcare professionals was limited despite its critical roles in healthcare practices [11, 12]. Promoting acceptance of e-health use requires an understanding of the relationships between e-health attributes as articulated by Rogers in the Diffusion of Innovation Theory which [13] proposes that individual’s response to new ideas influences the rate of diffusion of that idea through a social grouping [13]. The characteristics of an innovation determine its rate of adoption through five steps: a) being aware of the innovation and being able to gain some abstract idea of how it functions (knowledge); b) forming favourable or unfavourable attitudes toward the innovation (persuasion); c) engaging in activities that lead to a choice to adopt or reject the innovation (decision); d) deliberate action to put the innovation into use (implementation); and e) evaluating the results of the decision made toward the innovation. The process of adopting an innovation is complex [13, 14], and it involves a critical evaluation of five characteristics of the innovation, which include: relative advantage, compatibility, complexity, trialability and observability. In deciding to adopt e-health in professional practice, individual healthcare professionals might consider the following issues: a) Can e-health improve healthcare delivery? b) Does e-health technology fit well with the needs and current practices of healthcare professionals (compatibility)? c) Is e-health technology easy to use and understand (complexity)? d) Can e-health technology be tested or tried by healthcare professionals before making commitment to use it (trialability)? e) Can individual healthcare professionals see the benefits of using e-health technology in professional practice at the workplace (observability)? [11, 15]. If an evaluation of the attributes of e-health leads to its approval, healthcare professionals might use the innovation to improve the quality of healthcare service delivery. In addition to the five characteristics of innovation proposed by Rogers, we introduced healthcare professionals’ ICT skill in the model. We hypothesized that ICT skills will positively influence the adoption of e-health. In our model the level of ICT use by healthcare professionals was considered as the dependent variable.

Northern Uganda has experienced war and armed conflict starting 1987 to 2006 [16]. The war resulted in serious social, economic and health problems; health infrastructures were destroyed, and the region experienced major disease outbreaks that included Ebola, Hepatitis E and Nodding Syndrome. Mental health problems and tuberculosis in association with HIV/AIDS have emerged as new and serious public health problems in the region. Health facilities are currently facing enormous challenges in general disease management and response to outbreaks. Health systems suffer from low pay, difficulties in retaining staff, leading to serious health workforce inadequacy and poor resources to improve quality of healthcare services [17]. The Ugandan Ministry of Health has installed ICT equipment to address some of these challenges [17]. However, it is expected that the use of e-health can take shape only if healthcare professionals have positive attitudes in using ICT tools [7, 18–21]. Studies in developing countries have highlighted the following drawbacks: lack of knowledge about ICT; unreliable ICT equipment; high cost of ICT; low level of skills of potential users; technology compatibility; and limited access to ICT as challenges hindering ICT use [1, 22]. Further more, lack of ICT infrastructure, access, skills and reluctance to use e-health in healthcare services have been reported as challenges in the use of ICT [18, 21]. Rigorous data collected on the effectiveness of e-health in developing countries are still few [23] and studies have failed to answer questions about the contribution of e-health in facilitating healthcare services [3]. It is therefore not known if attitudes and ICT skills influence e-health adoption by healthcare professionals. This study aimed to determine the relationship between healthcare professionals’ attitudes toward e-health.

## Methods

### Study design and sites

We used a cross-sectional survey design. Sixty-eight medical doctors in three government hospitals and four private hospitals in Northern Uganda participated. Hospitals in this study is defined as “a registered healthcare facility, public or private organization, profit or not for profit, devoted to providing curative, disease prevention, health promotion and rehabilitative care through outpatient, inpatient and community health service” [17].

### Population and sample

Medical doctors, used interchangeably with healthcare professionals in this study, meant all doctors registered and working with the participating hospitals in various capacities such as trainee medical officers on internship, Medical Officers, Registered Medical Officers Special Grade, Specialists, Consultants and Senior Consultants. Though we intended to use Krejcie and Margan Table [24] for sample size determination, we included all medical doctors in the seven hospitals in Northern Uganda due to small numbers of potential respondents. For ethical reasons, hospitals were coded arbitrarily with letters A to G see Table 1.

**Table 1:** Respondents by hospital

	Actual number	Number reached	Percentage reached
Hospital			
A [PR]	5	3	60 %
B [GO]	2	1	50 %
C [PR]	7	6	85.7 %
D [PR]	47	32	68.1 %
E [PR]	8	8	100 %
F [GO]	2	2	100 %
G [GO]	18	16	88.9 %
Response rate			76.4 %

Note. *GO* Government hospital, *PR* Private hospital

### Ethical consideration

The Institutional Research Ethics Committee of Lacor Hospital and Uganda National Council for Science and Technology (UNCST) provided ethical approval with clearance numbers 038/10/13 and SS 3365 respectively. We obtained additional permission to access participants from the offices of Hospital Directors, and each respondent signed a written informed consent before we administered the questionnaire. Four Research Assistants were trained on research ethics, data collection procedures, questionnaire handling, record keeping and data protection. The first two authors supervised the Research Assistants throughout the data collection exercise.

### Instrument and procedures

A structured self-administered questionnaire designed for the study was pretested on a group of fifth year medical students before it was distributed to research participants. The questionnaire elicited information about background of doctors and assessed self-reported level of ICT skills and use as well as perception of doctors about ICT attributes. Questions elicited data on background characteristics of hospitals and respondents including participants’ gender, age and rank in the hospitals. Respondents’ level of ICT use was measured on a 5 point Likert type scale that ranged from “1 = very low” to “5 = very high”. Level of ICT skills was measured on a 5 point Likert type scale that ranged from “1 = very poor” to “5 = very good”. The perceived ICT attributes of relative advantage, compatibility, complexity, trialability and observability were rated on a 5 point Likert scale that ranged from “1 = strongly disagree” to “5 = strongly agree”. Scores for all statements for ICT use, ICT skills, and items for each perceived ICT attribute were averaged to create the specific “ICT use” and “ICT skills” mean score, and “perceived ICT attributes” mean score. Cronbach’s alpha was used to measure the internal consistency of each attribute in the questionnaire see Table 2.

**Table 2:** Internal consistency of questionnaire

Variable	Number of item	Cronbach’s alpha value
Level of ICT use	18	.930
Level of ICT skills	18	.944
Relative advantage	7	.917
Compatibility	4	.842
Complexity	5	.445
Trialability	5	.776
Observability	4	.820

Cronbach’s coefficient alpha is the most popular and generally applicable method of measuring reliability of a Likert scale [25, 26]. Most of the alpha values are greater than 0.7 indicating acceptable range [25, 26] except construct complexity with value below 0.7. It has been argued that when dealing with psychological constructs [in this study attitudes of medical doctors towards ICT attributes], “values below 0.7 may be expected because of the diversity of constructs being measured” [[27], p.120].

## Results

### Background information

As indicated in Table 3, Sixty-eight doctors responded to the survey (response rate of 76.4 %). The total percentage of medical doctors in Northern Uganda has been estimated as 7 % compared to other part of the country [17, 28, 29]. Of the respondents, majority 76.5 % were male and 23.5 %, female. This was expected as more than 77 % of medical doctors in Uganda are male [17]. The majority of respondents (48.5 %) were aged 31–40 years.

**Table 3:** Background information of respondents

Variable	Frequency	Percentage
Gender		
Female	16	23.5
Male	52	76.5
Age group		
21–30 Years	29	42.6
31–40 Years	33	48.5
41–50 Years	4	5.9
above 50 years	2	2.9
Rank		
Internship doctor	28	41.2
Medical officer	23	33.8
Medical officer special grade	3	4.4
Specialist (registrar)	12	17.6
Senior consultant	2	2.9

Three-quarters of medical doctors were in their junior stage of medical profession.

### Access to computer and Internet by healthcare professionals

Table 4 indicates that Only thirty-nine (57.4 %) healthcare professionals reported access to a computer and thirty-three (48.5 %) respondents had access to Internet facilities. Proportionately more females (10, 62.5 %) than males (29, 55.8 %) reported access to a computer.

**Table 4:** Access to computer and internet

	Access to computer	Access to internet		
Gender	Yes	No	Yes	No
Female	10 (62.5 %)	6 (37.5 %)	10 (62.5 %)	6 (37.5 %)
Male	29 (55.8 %)	23 (44.2 %)	23 (44.2 %)	29 (55.8)
Total	39 (57.4)	29 (42.6)	33 (48.5 %)	35 (51.5 %)
Age				
21–30 years	16 (55.2 %)	13 (44.8 %)	14 (48.3 %)	15 (51.7 %)
31–40 years	20 (60.6 %)	13 (39.4 %)	17 (51.5 %)	16 (48.5 %)
41–50 years	1 (25.0 %)	3 (75.0 %)	0 (0.0 %)	4 (100.0 %)
Above 50 years	2 (100.0 %)	0 (0.0 %)	2 (100.0 %)	0 (0.0 %)
Total	39 (57.4 %)	29 (42.6 %)	33 (48.5 %)	35 (51.5 %)
Rank				
Interns doctor	11 (39.3 %)	17 (60.7 %)	8 (28.6 %)	20 (71.4 %)
Medical officer	17 (73.9 %)	6 (26.1 %)	18 (78.3 %)	5 (21.7 %)
Medical officer special grade	3 (100.0 %)	0 (0.0 %)	2 (66.7 %)	1 (33.3 %)
Specialist	6 (50.0 %)	6 (50.0 %)	4 (33.3 %)	8 (66.7 %)
Senior consultant	2 (100.0 %)	0 (0.0 %)	1 (50.0 %)	1 (50.0 %)
Total	39 (57.4 %)	29 (42.6 %)	33 (48.5 %)	35 (51.5 %)

### Health professionals’ attitude towards e-health attributes

Our findings indicated that healthcare professionals had moderate to strong positive attitudes toward ICT with a “relative advantage” mean of 4.3; “compatibility” mean of 3.8; “trialability” mean of 3.2; and “observability” mean of 3.5 as summarized in Table 5 (see Appendix for details).

**Table 5:** Attitudes toward e–health attributes

E–health attitudes	Total mean score
ICT Relative advantage	4.3
Compatibility	3.8
Complexity	2.9
Trialability	3.2
Observability	3.5

### Healthcare professionals’ level of ICT use

As indicated in Table 6, Respondents’ levels of use of e-health facilities varied across all the ICT facilities and tools available in the hospitals.

**Table 6:** Level of ICT use

ICT Facilities and tools in hospital	Very low	Low	Moderate	High	Very high	Mean score
	(%)	(%)	(%)	(%)	(%)	
Computer (PCs)	16.2	13.2	29.4	20.6	20.6	3.16
Printer	33.8	16.2	30.9	13.2	5.9	2.41
Fax machine	73.5	11.8	4.4	5.9	4.4	1.56
Mobile phone	7.4	4.4	10.3	26.5	51.5	4.10
Tablet computer (touch screen)	48.5	14.7	11.8	10.3	14.7	2.28
Digital camera	35.3	23.5	16.2	8.8	16.2	2.47
Computerize sensor	63.2	17.6	10.3	7.4	1.5	1.66
Body scanner	63.2	16.2	11.8	7.4	1.5	1.68
Computerize databases	47.1	8.8	20.6	13.2	10.3	2.31
Flask disk/memory sticks	16.2	8.8	20.6	26.5	27.9	3.41
CD-ROMs/DVDs	26.5	13.2	19.1	19.1	22.1	2.97
E-mail	13.2	13.2	22.1	26.5	25.0	3.37
E-journals	26.5	17.6	26.6	13.2	16.2	2.75
E-books	25.0	17.6	23.5	16.2	17.6	2.84
Internet	13.2	14.7	17.6	20.6	33.8	3.47
Microsoft access	22.1	20.6	32.4	8.8	16.2	2.77
Microsoft excel	19.1	16.2	27.9	19.1	17.6	3.00
Microsoft PowerPoint	10.3	13.2	19.1	30.9	26.5	3.50
Total mean score						2.76

The top five ICT facilities and tools that healthcare professionals used were the mobile phone (mean 4.10), Microsoft PowerPoint (mean 3.50), Internet (mean 3.47), flash disk/ memory sticks (mean 3.41) and e-mail (mean 3.37). In reality our findings revealed that the most popular ICT equipment among doctors in Northern Uganda were mobile phones and computers. It is surprising that body sensor scanner was rated very low though nearly all the participating hospitals in Northern Uganda had at least one ultra-sound scanner in use.

### Healthcare professionals’ level of ICT skills

As indicated in Table 7, Respondents’ levels of ICT skills varied across all the ICT facilities and applications available in these hospitals.

**Table 7:** Levels of ICT skills

ICT Facilities and applications	Very poor	Poor	Fair	Good	Very good	Mean score
	(%)	(%)	(%)	(%)	(%)	
Using computer (PCs)	4.4	0.0	13.2	42.6	39.7	4.13
Using printer	11.8	10.3	16.2	36.8	25.0	3.53
Using fax machine	39.7	26.5	17.6	8.8	7.4	2.18
Using mobile phone	2.9	1.5	2.9	22.1	70.6	4.56
Using tablet computer (touch screen)	10.3	10.3	19.1	26.5	33.8	3.63
Using digital camera	8.8	7.4	16.2	25.0	42.6	3.85
Using computerize sensor	32.4	35.3	17.6	11.8	2.9	2.18
Using body scanner	35.3	33.8	16.2	11.8	2.9	2.13
Using microsoft word	4.4	0.0	8.8	36.8	50.0	4.28
Using flask disk/memory sticks	5.9	0.0	11.8	25.0	57.4	4.28
Using CD-ROMs/DVDs	8.8	0.0	10.3	33.8	47.1	4.10
Using E-mail	2.9	2.9	13.2	26.5	54.4	4.27
Using E-journals	4.4	8.8	22.1	30.9	33.8	3.81
Using E-books	8.8	7.4	22.1	27.9	33.8	3.71
Using microsoft access	7.4	14.7	38.8	23.5	20.6	3.35
Using microsoft excel	5.9	8.8	33.8	30.9	20.6	3.52
Using microsoft PowerPoint	5.9	1.5	19.1	27.9	45.6	4.06
Using internet	2.9	4.4	13.2	25.0	54.4	4.24
Total mean score						3.66

Results revealed, however, that the level of skills for actual use of ICT tools was slightly better than the corresponding levels of use of the tools. As in the level of access, respondents’ mean scores were once high again for the mobile phones, computer and its accessories. It is not immediately clear if the observed differences of the level of use and actual use of ICT tools were a reflection of the level of access to the equipment in the workplace of doctors in Northern Uganda.

### Predicting the level of ICT use by healthcare professionals

As in Table 8, a standard multiple regression was performed between level of ICT use by healthcare professionals as the dependent variable and level of ICT skills, relative advantage, compatibility, complexity, trialability and observability as independent variables. The results revealed that 41.8 % of the variance in ICT use was predicted by the combined influence of observability, relative advantage, compatibility level of ICT skills, trialability and complexity as in Table 7.

**Table 8:** Multiple regression of ICT attributes and ICT skills on ICT use

Predictors	Coefficients	Standardized coefficients	t	Sig.	Collinearity statics		
	B	Std. error	Beta			Tolerance	VIF
(Constant)	33.079	13.045		2.536	.014		
Level of ICT skills	.522	.117	.468	4.470	.000	.872	1.147
Relative advantage	.601	.398	.194	1.511	.136	.577	1.733
Compatibility	-.621	.617	-.137	−1.006	.318	.516	1.938
Complexity	-.019	.470	-.004	-.040	.968	.924	1.083
Trialability	-1.017	.403	-.279	−2.522	.014	.779	1.284
Observability	-.458	.470	-.114	-.976	.333	.697	1.436

a. Dependent Variable: ICT Use

However the actual attributes that contributed to healthcare professionals’ use of ICT equipment and tools were the level of ICT skills that the professionals (*p = 0.000*) had, and the influence of trialability (*p = 0.014*), which together accounted for 41.8 % of the variance in the use of e-health. Thus the level of ICT skills among healthcare professionals appeared to be the most important and significant predictor of ICT use in the workplace.

## Discussion

### Health professionals’ attitude towards e-health attributes

Majority of healthcare professionals generally had positive attitude towards e-health attributes. Healthcare professionals in this study appeared to consider e-health as a) improvement over the current practices in medical field, b) e-health technology fitted well with the need and current practices of healthcare professionals, c) e-health technology was easy to use, d) e-health technology could be tested or tried and e) it was easy to observe and learn about the use of e-health facilities in the workplace. A number of studies have assessed attitudes of healthcare professionals towards ICT [30–34]. Related studies claimed that healthcare professionals had positive attitudes toward ICT [21, 33, 35, 36]. Our study found generally positive attitudes towards ICT which is similar to that of Loh et al. [33], Woodward et al. [21], Kipturgo et al. [37], Gagnon et al. [32] and Zailani et al. [38]. The positive attitudes toward e-health attributes appear to suggest that ICT could offer benefits [39] in the delivery of health services in Northern Uganda.

### Healthcare professionals’ level of ICT use

Though there were three times as many male doctors as females, proportionately more female (62.5 %) healthcare professionals than males (44.2 %) reported having had access to a computer. The use of computer (60.6 %) and Internet (51.5 %) in the hospitals was proportionately more among relatively young healthcare professionals aged 21–40 years. Surprisingly, healthcare professionals above 50 years of age have all used computer 2 (100 %) and Internet 2 (100 %) compared with healthcare professionals aged between 41–50 years who reported less use computer (25 %) and Internet (0 %). Though these observations are of interest their significance is limited by the very few numbers of various groups of healthcare professionals that participated in the study. Majority of medical officers have access to computer (73.9 %) and Internet 18 (78.3 %). Surprisingly, majority of Medical Officers Intern did not report access to computer 17 (60.7 %) and Internet 20 (71.4 %) in the hospitals. Internship is a period of intense practical and theoretical training and learning. Lack of access to computer and Internet facilities among this group of healthcare professionals is surprising and potentially undermines the quality of effective training and learning during internship. It is curious that more female doctors reported access to computers and Internet facilities than male doctors. It is not clear if this is a function of interest or ownership of e-health facilities among female and male doctors in Northern Uganda. Our findings suggest that access to computer and Internet facilities was related to rank of healthcare professionals with doctors aged more than 50 years reporting more access to ICT facilities. It is not immediately clear if this association between access to ICT facilities and Internet was a function of age or the respondents’ level of seniority and responsibility at work, which placed a lot of demand on the senior level doctors to keep abreast with the ever-changing knowledge base and practice patterns in the medical profession.

Overall, our findings show that the level of ICT use by healthcare professionals in the hospitals was low. The use of mobile phone was high among healthcare professionals, confirming claims that the use of mobile phones had made its way in the healthcare services [9, 30, 40]. Mobile phones are reported to help in remote diagnostic monitoring, data collection and health information dissemination over cellular networks [23, 41]. In addition the web-based application software in mobile phones can be configured to send and receive medical reports, pop up alerts any time at healthcare professionals’ convenient. The adoption of mobile phones by healthcare professionals was triggered by the inability of some health facilities to provide reliable electricity, Internet access coupled with the high cost of maintenance and the level of skills needed to use other ICT equipment [4]. Though a majority of health care professionals reported using the mobile phone, it was difficult to predict the extent to which mobile phones contribute to healthcare service [42]. The ICT facilities and tools that received less use in the hospitals could be a result of various reasons, which include lack of ICT facilities in the workplace, inadequate skills in operating ICT equipment, the specialized nature of the equipment, and the type of health facility. For examples, ICT tools such as the body scanners and computerised sensors are specialised and expensive tools, which may not be available and accessible to all healthcare professionals. Other equipment, such as fax machine may be considered insecure for transmitting patients’ information compared to the use of the more secure e-mail to send medical records. Given the overall positive attitude of healthcare professionals to e-health, it is important that more efforts be directed toward improving ICT skills training and building and maintaining ICT infrastructure to support healthcare service.

### Health professionals’ level of ICT skills

Our findings revealed a fair to moderate level of use of ICT skills as reported by other workers [35, 43]. This level of skill is, however, worrying considering the speed at which e-health is penetrating healthcare. The level of ICT skills demonstrated in this study is likely to be major hindrance to the use of ICT [1]. The self-reported level of ICT skills in this study reflects healthcare professionals’ confidence and some understanding of ICT applications in healthcare. It appears that ICT skills were better in the commonly available ICT tools and applications in hospitals. It was not surprising that the results indicated better levels of use of ICT tools that healthcare professionals have continuously used based on experience and self-learning. This is consistent with previous studies where facilities and applications, such as Internet, e-mail, word processor, PowerPoint and computer received higher levels of use among healthcare professionals [37, 44]. Despite good ICT skills in some of the ICT facilities, healthcare professionals had poor skills in using body scanner, computerised sensor and fax machine. We believe that ICT facilities and tools, which received less use in the hospitals, might be a result of lack of such tools in the hospitals, inadequate skills in operating them, the specialised nature of equipment and tools, and the type of healthcare facility. As a result, healthcare professionals have tended to move to the more secure and faster mobile phones and e-mail applications on smartphones or computers to send medical records among healthcare professionals and patients. Investing in ICT skill training guided by ICT training needs assessment might be appropriate in building ICT skills and use among healthcare professionals in Northern Uganda.

### Predicting ICT use from ICT attributes and skills

Our finding indicates that the level of ICT skills was the most important and statistically significant predictor of ICT use, as reported in other studies [35, 38, 45]. It might therefore be reasonable to suggest that improving healthcare professionals’ ICT skills might directly improve their level of ICT use by healthcare professionals in hospitals in Northern Uganda. Lack of ICT related skills constitutes serious barrier to ICT use [22]. Training to improve ICT skills of healthcare professionals should take a strategic approach tailored toward specific areas identified through hospital ICT needs analysis. Since trialability was the most important predictor of ICT use, the outcomes of skills development might be enhanced if hospital administrations in Northern Uganda provide opportunities for their healthcare personnel to learn from their peers at the workplace. Though the attributes of relative advantage, compatibility, complexity and observability also predicted ICT use in our study, other studies did not report the same result [20, 46]. It is possible that perceived ICT attributes may not be a good predictor of ICT use in healthcare settings in Northern Uganda; it is also possible that the small sample size in our study might not have provided sufficient data to enable us obtain different sets of results. However, it is also possible that factors other than those in the innovation theory contributed to the self-reported use of e-health among respondents. These factors might have been level of ICT skills, access to ICT, availability of ICT, and time available for healthcare professionals to access and use ICT tools in the workplace.

### Limitations

Our major limitation was the small sample size, which we could not control, as the number of healthcare professionals in Northern Uganda is very limited indeed due to the region’s remoteness from the Capital City of Uganda, and the long history of armed conflict that affected the region for more than two decades. It would be useful to repeat our study in the whole country to determine the attitudes of a larger sample of doctors in more facilities and regions than we were able to achieve. We used quantitative method with predetermined responses, which might not have permitted respondents’ views to provide varied but useful qualitative information about their attitudes toward e-health and its use in health service delivery. Accordingly, we recommend the additional use of qualitative method to determine the attitudes of healthcare professionals toward e-health, from the perspective of Diffusion of Innovation Theory [47].

## Conclusion

E-health is a new and specialized concept in healthcare delivery in Uganda, and its application has not been deliberately addressed to improve individual- and population-level health. However our respondents reported fairly positive attitudes toward e-health despite the multitude of structural and systemic difficulties. Based on our findings, there is hope for e-health to be fully integrated in the healthcare system in Northern Uganda.

## Appendix

**Table 9:** Attitudes of doctors towards e-health attributes

ICT Relative advantage	SD	D	NS	A	SA	Mean score
	*%*	*%*	*%*	*%*	*%*	
Using ICT enables me accomplish medical task more quickly	1.5	7.4	2.9	29.4	58.8	4.4
Using ICT improves the quality of medical work I do	0.0	4.4	7.1	22.1	66.2	4.5
Using ICT make me do my medical work easily	0.0	5.9	4.4	38.2	51.1	4.4
Using ICT make me improve my job performance	1.5	1.5	1.5	36.8	58.8	4.5
Using ICT enhance my effectiveness on my job	1.5	1.5	4.4	33.8	58.8	4.5
Using ICT gives me greater control over my work	4.4	7.4	16.2	33.8	38.2	3.9
Using ICT increases my work productivity	2.9	11.8	4.4	30.9	50.0	4.1
Total mean score						4.3
Compatibility						
Using ICT is compatible with all aspects of my work	5.9	11.8	17.6	38.2	26.5	3.7
Using ICT is completely compatible with my current situation	5.9	10.3	16.2	42.6	25.0	3.7
I think ICT I used fits well with the way I like to work	4.4	10.3	7.4	52.9	25.0	3.8
Using ICT fits well into my work style	2.9	7.4	8.8	48.5	32.4	4
Total mean score						3.8
Complexity						
I believe that using ICT is cumbersome	27.9	38.2	13.2	11.8	8.8	2.4
Using ICT require a lot of mental effort	23.5	36.8	8.8	19.1	11.8	2.6
Using ICT is often frustrating	26.5	25	19.1	13.2	16.2	2.7
I believe that it is easy to make ICT do what I want it to do	7.4	10.3	10.3	51.5	20.6	3.7
Learning to operate ICT is easier for me	5.9	20.6	17.6	42.6	13.2	3.4
Total mean score						2.9
Trialability						
I’ve had a great deal of opportunity to try ICT applications	11.8	25	10.3	36.8	16.2	3.2
I know where I can go to satisfactorily try out ICT	7.4	14.7	22.1	38.2	17.6	3.4
I always try out ICT applications before using it	8.8	14.7	10.3	52.9	13.2	3.5
I use ICT on a trial basis enough to see what it could do	23.5	23.5	22.1	26.5	4.4	2.6
I do not have to take very much effort to try out ICT	10.3	29.4	16.2	29.4	14.7	3.1
Total mean score						3.2
Observability						
I have seen what other hospital staff do with ICTs	7.4	7.4	5.9	55.9	23.5	3.8
In the hospital, I see ICT being used for many tasks	8.8	13.2	5.9	42.6	29.4	3.7
ICT is very visible in the hospital where I work	13.2	25	14.7	29.4	17.4	3.1
It is easy to observe people using ICT in the hospital	14.7	13.2	16.2	39.7	16.2	3.3
Total mean score						3.5
